# Supplementary material for: S-WD-EEMD: A hybrid framework for imbalanced sEMG signal analysis in diagnosis of human knee abnormality
Source: PLoS One. 2024 May 31;19(5):e0301263. doi: 10.1371/journal.pone.0301263 (PMC11142505; doi:10.1371/journal.pone.0301263)
Supplement: Attachment — Submitted filename: Review_PONE.pdf [file pone.0301263.s001.pdf]

Manuscript Number: PONE-D-23-43284

AUTHORS' RESPONSES TO THE REVIEWERS

The authors would like to express their gratitude to the Reviewers for spending their valuable time and providing various insightful recommendations to further improve the manuscript submitted to the "PLOS ONE" journal.

The authors have attempted their best to incorporate all suggestions made by the Reviewers and answer all queries made by them. The changes are indicated in the revised manuscript in red colored text.

Reviewer 1:

Comments:

Q1. The authors are claiming a very high-performance accuracy of 99%, how the authors handle overfitting issue of the model to validate the generalization of the model.

R1. The authors are extremely grateful for this important suggestion. The results are re-examined with 10-fold stratified cross-validation to handling the overfitting issue. The accuracy of the proposed S-WD-EEMD model is slightly decreased from 90.2% to 90%. The changes of the performance parameters are **updated in Table 3 (Page no. 14).** Still our proposed model is performing better than the other individual models and the previous studies.

In addition to reporting high accuracy, we also evaluated our model using other metrics such as precision, recall, F1-score, and ROC curve. These metrics provide a more comprehensive understanding of the model's performance across different aspects and help in verifying its generalization ability beyond just accuracy.

Q2. To prove the effectiveness of the model, the thorough comparison of the proposed model with existing studies are needs to be performed.

R2. In the updated manuscript, the authors compared their model with existing studies that utilized the same dataset in the Table 4. From this table, we can see that the proposed technique performs better in identifying individuals with knee abnormalities as compare to the previous studies.

Table 4. Assessing the effectiveness of the proposed methodology by comparing its performance with existing literature studies utilizing similar datasets (in %).

Approach	SMOTE + ET [43]	ADAYSN + ET [43]	SVM SMOTE + ET [43]	XGBoost [69]	Proposed Method
**Accuracy**	93.1	92.2	93.2	96	**99.0**
**F-Score**	85.3	84.2	85.1	94	**98.4**

In addition to this table, the authors have added the following text to explain the contents of the table **(Page no. 17-18, Line no.523-531)** in the revised manuscript.

"Very few studies have been published regarding the detection of knee abnormalities in the literature using the same dataset. In our previous research, we applied various oversampling approaches such as SMOTE, ADAYSN, and SVM SMOTE, and evaluated their performance using the Extra Tree machine learning classifier as presented in Table 4. Rani et al. also utilized the same dataset to classify subjects with knee abnormalities using machine learning approaches. Their XGBoost classifier achieved an accuracy of 96% with an F-Score of 94%. Table 4 compares our suggested model's performance with previous studies on the same dataset. Our findings suggest that the proposed technique performs better in identifying individuals with knee abnormalities."

Q3. The discussion is not clear, authors are advised to provide the advantages and limitations of the proposed model.

R3. Discussion section has been improved in the revised manuscript as per the suggestion of reviewer. The authors added the advantages and limitations of the proposed model. The authors have added a discussion on the advantages and limitations of the proposed model. The following text has been added in the revised manuscript to elucidate the advantages and disadvantages of the proposed model **(Page No. 15, lines 445-452).**

"WD-EEMD excels in extracting richer features from time series data in contrast to traditional methods, effectively capturing both time and frequency domain characteristics. Integrating SMOTE with WD-EEMD ensures the generation of synthetic samples that align with the local data characteristics, thereby enhancing the diversity and relevance of the samples. This augmentation contributes to improved generalization and heightened classification accuracy, especially in scenarios with imbalanced datasets. However, a notable limitation of this hybrid approach is its potential to substantially escalate computational demands due to the iterative processes involved in both techniques."

Q4. For the reader concerns, the recent predictors should be cited such as iAFP-gap-SMOTE, and AIPs-SnTCN in SMOTE section. additionally, iAFPs-EnC-GA model should be cited and discuss in the wavelet transformation section.

R4. As per the suggestions, the authors have cited the suggested relevant models in their introduction section of the updated manuscript **(See Reference 42, 53).**

Q5. The conclusion needs more improvements in term of contribution, advantages, achieved results than existing model.

R5. The conclusion section has been improved in the updated manuscript **(Page no. 18, Line no. 533-570).** The authors added the contribution and the advantages of the proposed model and shows the improvement of the proposed model with the existing approaches. The proposed hybrid approach combining Wavelet Decomposition (WD), Ensemble Empirical Mode Decomposition (EEMD), and Synthetic Minority Over-sampling Technique (SMOTE) can potentially be extended to other medical applications characterized by imbalanced data.

Reviewer 2:

Comments:

Q1. The feature extraction methods are very shortly explained, I suggest adding more details to the readers.

R1. As per the suggestions of the reviewer, the section of the feature extraction methods are improved in the updated manuscript. The authors added the reason of the feature extraction model and what are the impact of the feature extraction during the machine learning. The following text has been added in the updated manuscript **(Page no. 8, Line no. 275-280).**

"The conventional approach to applying a machine learning model relies on handcrafted features. This method has garnered widespread acceptance within the research community and has yielded remarkable results on numerous widely recognized public datasets. It involves extracting meaningful features from a signal and subsequently employing a generic machine learning classifier, such as a Support Vector Machine (SVM) or Linear Discriminant Analysis (LDA), for classification purposes."

Q2. The optimal parameters selected for training the machine learning models should be provided in the form of a table.

R2. The parameters selected for the training of the machine learning models is added in the form of table in the updated manuscript in Table 2 (Page no. 13).

Table 2. Parameters of Machine Learning Models

Model	Parameter
ID3	criterion='entropy'<br>min_samples_split=2<br>min_samples_leaf=1<br>min_weight_fraction_leaf=0.0<br>min_impurity_decrease=0.0<br>ccp_alpha=0.0
CART	criterion='gini'<br>min_samples_split=2<br>min_samples_leaf=1<br>min_weight_fraction_leaf=0.0<br>min_impurity_decrease=0.0<br>ccp_alpha=0.0
Bagging	n_estimators=10<br>max_samples=1.0<br>max_features=1.0
ET	n_estimators=100<br>criterion='gini'<br>min_samples_split=2<br>min_samples_leaf=1<br>min_weight_fraction_leaf=0.0<br>max_features='sqrt'<br>min_impurity_decrease=0.0<br>verbose=0<br>ccp_alpha=0.0
SVM	C=1.0<br>kernel='rbf'<br>degree=3<br>gamma='scale'<br>tol=0.001<br>cache_size=200<br>max_iter=-1<br>decision_function_shape='ovr'
GBC	loss='log_loss'<br>learning_rate=0.1<br>n_estimators=100<br>subsample=1.0<br>criterion='friedman_mse'<br>min_samples_split=2<br>min_samples_leaf=1<br>min_weight_fraction_leaf=0.0<br>max_depth=3<br>min_impurity_decrease=0.0<br>validation_fraction=0.1<br>tol=0.0001<br>ccp_alpha=0.0
RF	n_estimators=100<br>criterion='gini'<br>min_samples_split=2<br>min_samples_leaf=1<br>min_weight_fraction_leaf=0.0<br>max_features='sqrt'<br>min_impurity_decrease=0.0<br>ccp_alpha=0.0
MLP	hidden_layer_sizes=(100)<br>solver='adam'<br>alpha=0.0001<br>learning_rate_init=0.001<br>power_t=0.5<br>max_iter=200<br>tol=0.0001<br>momentum=0.9<br>validation_fraction=0.1<br>beta_1=0.9<br>beta_2=0.999<br>epsilon=1e-08<br>n_iter_no_change=10<br>max_fun=15000

Q3. What should be the real-life applications developed based on the proposed model, authors should discuss.

R3. While the authors had already added real-life application in the introduction section, we have also now highlighted the applications of the proposed model in the discussion and conclusion. The following text has been added in the updated manuscript (Page no. 18, Line no. 555-560, Line no. 565-570).

"In summary, the proposed model can be used to identify individuals with potential knee injuries (anterior cruciate ligament and meniscus damage), or possibly those predisposed to knee injuries depending on how advanced the change in neuromuscular EMG functioning is relative to the tissue structural damage. These individuals could then be referred for clinical examination and either prevention or rehabilitation, depending on level of injury.

Furthermore, the proposed hybrid approach combining Wavelet Decomposition (WD), Ensemble Empirical Mode Decomposition (EEMD), and Synthetic Minority Over-sampling Technique (SMOTE) can potentially be extended to other medical applications characterized by imbalanced data. This suggests its potential for addressing similar challenges in diverse medical contexts, thereby expanding its applicability and relevance beyond the scope of this study."

Q4. How many features were extracted after applying proposed methods, authors should discuss in the paper.

R4. Nine features were extracted from the proposed methods. The authors added following lines in the updated manuscript (Page no. 8, Line no. 284-286).

"To improve the accuracy and reduce the computational latency of a classifier, nine time domain features are extracted from the signal. These features are used as inputs to the classifier model."

Q5. In order to show the effectiveness of the proposed model, the ROC analysis of the training models is highly recommended.

R5. ROC curves of the machine learning models are added in the updated manuscript as Fig 4 (Page no. 16).

In addition to this figure, the authors have added the following text to explain the plotted curve **(Page no. 17, Line no.505-522)** in the revised manuscript.

"To support the findings presented in Table 3 and provide additional insights, a Receiver Operating Characteristic (ROC) analysis was conducted and illustrated in Fig 4. ROC analysis serves to validate the efficacy of a statistical machine learning model for a given problem by plotting a curve representing the true positive rate against the false positive rate. Each point on the ROC curve corresponds to an instance of a confusion matrix. Ideally, a model is considered 100\% accurate if its ROC curve exhibits a point in the top-left corner of the plot. Observing Fig 4, it is apparent that before applying SMOTE, when the data exhibits a non-uniform distribution, the ROC curves for all models slightly shift towards the false positive region. This suggests a bias towards a specific class due to data imbalance, a common challenge in learning with imbalanced data. However, upon applying SMOTE, there is a noticeable shift towards the ideal point (0,1) on the ROC plot, indicating improved model performance. Furthermore, the simultaneous application of WD and EEMD pre-processing with SMOTE leads to further improvement in model performance, as evidenced by the ROC curve shifting closer to the ideal point. This underscores the efficacy of these pre-processing techniques in enhancing model identification across all testing data instances. These findings emphasize the critical importance of addressing data imbalance to achieve satisfactory classification algorithm performance. Moreover, the utilization of WD-EEMD pre-processing techniques not only mitigates outliers but also brings numerical features within a considerable range, thereby facilitating enhanced classification."

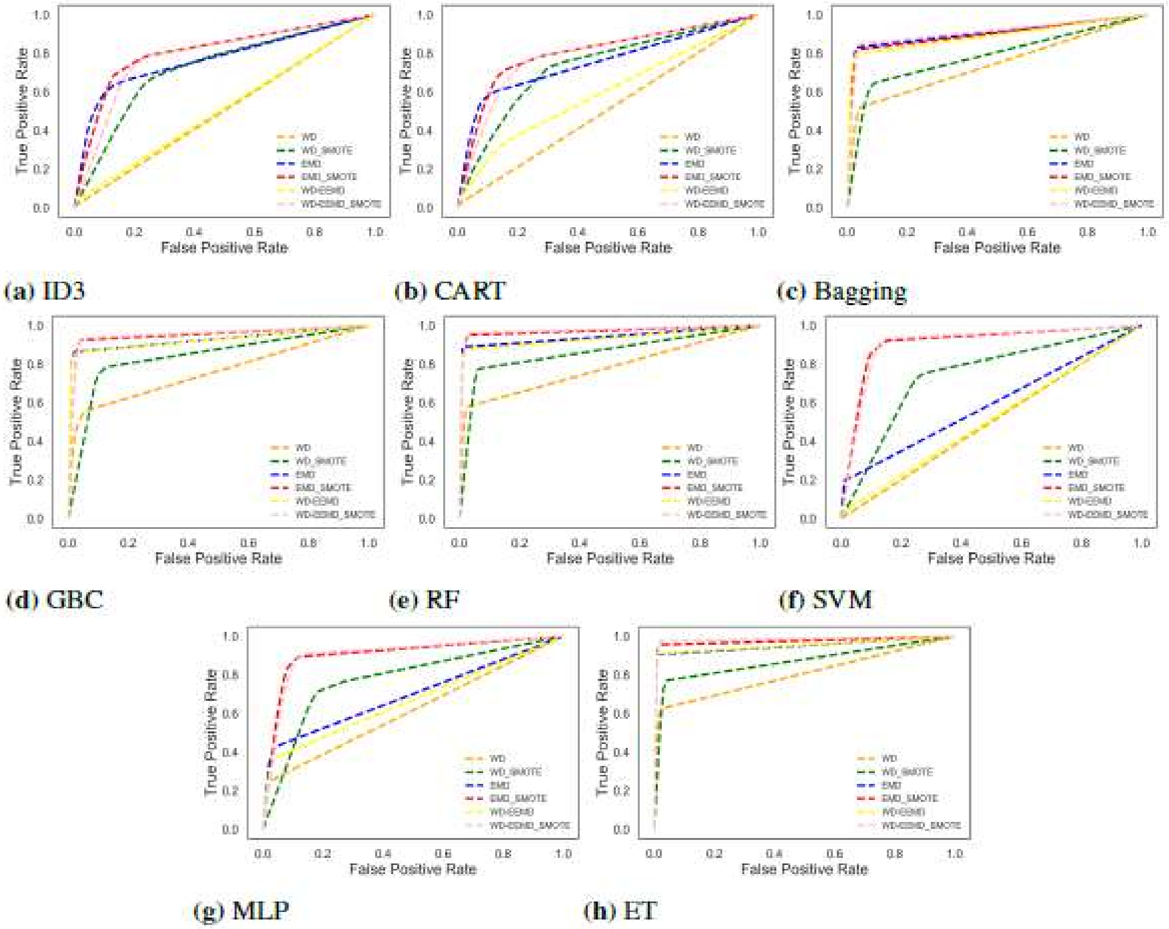

Fig 4. ROC Curve.

Q6. The recent computational models such as 10.1109/TNSRE.2016.2636367, 10.3389/fgene.2020.539227, 10.1109/ACCESS.2021.3062291.

R6. As per the suggestions, the authors have cited these suggested relevant models in their updated manuscript **(See Ref. 54, 55)**.

The proper care has been taken to pay attention to all the concerns raised by the reviewers and modifications have been made accordingly in the revised manuscript.
